# Montenegro makes important strides towards achievement of the SDGs

**DOI:** 10.1093/eurpub/ckaa030

**Published:** 2020-05-11

**Authors:** Ministry of Health, Montenegro, Oliver Schmoll, Enkhtsetseg Shinee, Mina Brajovic, Bettina Menne, Francesco Zambon, Leda Nemer

**Affiliations:** c1 Ministry of Health, Podgorica, Montenegro; c2 Water and Climate Programme, WHO European Centre for Environment and Health, Bonn, Germany; c3 Montenegro Country Office, WHO Regional Office for Europe, Copenhagen, Denmark; c4 Sustainable Development and Health, Division of Policy and Governance for Health and Well-being, WHO Regional Office for Europe, Copenhagen, Denmark; c5 Investment for Health and Development in Healthy Settings, WHO European Office for Investment for Health and Development, Venice, Italy

## Abstract

In 2018, Montenegro took an important step towards ratification of the Protocol on Water and Health to the 1992 Convention on the Protection and Use of Transboundary Watercourses and International Lakes. A multisectoral national consultation provided a forum where national stakeholders could assist in related decision-making. The Protocol is the first and only multilateral legal agreement linking sustainable water management and the prevention, control and reduction of water-related diseases in the pan-European region. It was adopted in 1999 at the Third Ministerial Conference on Environment and Health in London and entered into force in 2005 as legally binding for the ratifying countries. To date, 26 countries have ratified it, covering about 60% of the population of the pan-European region. Montenegro is on the way to becoming the next country to ratify it and has used it as an instrument to strengthen national action towards progressively reaching regional and global WASH-related commitments, specifically in relation to SDG 3 (good health and well-being), SDG 6 (clean water and sanitation) and the Ostrava Declaration on Environment and Health (2017).

## The issue

Water scarcity, poor water quality and inadequate sanitation negatively impact food security, livelihood choices and educational opportunities in the WHO European Region. In Montenegro, about 96% of the total population use at least basic sanitation services (improved sanitation facilities that are not shared with other households) and 97% use basic and safely managed drinking-water services. However, large differences exist between rural and urban areas. Data are not available on how many of the people living in rural areas use safely managed drinking-water and sanitation.^1^ In urban areas, while drinking-water and sanitation policies and plans are in place, polices and plans addressing rural drinking-water and sanitation are lacking. Interim results from the 2018–19 Global Analysis and Assessment of Sanitation and Drinking-Water (GLAAS) country survey^2^ highlights the presence of an important type of inequity in Montenegro, indicating the need to improve the governance of water and sanitation and make it accessible to all, paying special attention to the rural areas. They also point to the necessity to strengthen national capacity for monitoring progress towards the sustainable development goal (SDG)-6 targets^3^ related to the provision of safely managed services to ensure no one is left behind. The WHO Roadmap to implement the 2030 Agenda for Sustainable Development, building on Health 2020, the European policy for health and well-being^4^ places national multisectoral action and international cooperation for health at its centre. The implementation of multilateral environment agreements, such as the Protocol,^5^ will pave the way for action to achieve the SDGs.^3^

## The process

The small countries initiative (SCI; is a platform through which the 11 Member States in the WHO European Region with populations of <2 million are able to share their experiences in implementing Health 2020, the 2030 Agenda for Sustainable Development and the WHO roadmap to implement the 2030 Agenda, building on Health 2020. The countries participating in the Initiative are Andorra, Cyprus, Estonia, Iceland, Latvia, Luxembourg, Malta, Monaco, Montenegro, San Marino and Slovenia. Yearly high-level meetings result in soft commitments (statements, manifestos etc.) by member countries to take action on a number of pertinent issues affecting small countries.) Iceland Statement on, ‘Ensuring safe and climate-resilient water and sanitation (2018),’^6^ was an important trigger in initiating the process towards ratifying the Protocol.^5^ The Statement^6^ motivated Montenegro to embark on an inclusive ‘consultative process’ in which representatives of different key ministries, the Parliament and civil society—all of which share responsibility for WASH—are actively engaged. The process also provided an ideal opportunity to introduce the policy context relating to the Protocol^5^ (the SDGs, the Ostrava Declaration^3^^,^^7^), as well as its requirements, and to compare related experiences from neighbouring countries. The ministries for health, agriculture, sustainable development and tourism are represented in the consultative process, which is led by the Ministry of Health.

## Achievements to date

A key outcome of the consultative process is the agreement to establish a national Protocol working group responsible for: baselining; setting and revising targets and defining indicators; planning action, including timing, responsibilities and budgeting; communicating with the general public; overseeing and tracking progress towards achievement of the targets; and coordinating mandatory reporting exercises as specified in the Protocol.^5^ It is envisaged that the working group will comprise representatives of ministries/institutions responsible for water, sanitation, health, education, finance, environmental protection, sustainable development, tourism, agriculture, sanitary inspection, human rights and climate change, as well as service providers, civil society and municipalities.

Additional steps in Montenegro’s progress towards ratification of the Protocol^5^ will include:

- establishing a national baseline analysis of the situation pertaining to WASH;
- setting national priority targets aligned with SDGs 3 and 6,^3^ the Ostrava Declaration on Environment and Health,^7^ and the commitments of the Iceland Statement^6^;
- supporting action towards the accession of Montenegro to the European Union (EU; targets set under the Protocol should reflect the national socioeconomic and environmental health conditions and priorities for improving needs in the WASH domain);
- formulating an action plan towards implementation of the national targets and leveraging opportunities for mobilizing resources to support it;
- engaging with civil society in setting targets and implementing action towards their achievement by ensuring that appropriate provisions for public participation are included in a transparent and fair framework and that the outcome of public participation is taken into consideration.

## A special focus on persisting inequalities

One of Montenegro’s targets will pay special attention to tackling the persisting inequalities in access to water and sanitation services, and particularly to closing the rural–urban gap in access to safely managed and climate-resilient water and sanitation services. It will also focus on the provision of safe and sustainable water, sanitation and hygiene services in schools with a view to achieving better health and educational outcomes. Reaching this target area will call for strengthening national WASH surveillance systems in schools and establishing national indicators in accordance with SDGs 4 and 6,^3^ as recommended by the WHO/UNICEF Joint Monitoring Programme for Water Supply, Sanitation and Hygiene.^1^

## Conclusion

The Protocol^5^ has provided Montenegro with a practical framework for delivery of action towards achievement of the various SDGs related to WASH.^3^ This includes practical tools for use in addressing inequalities in access to safely managed and climate-resilient water and sanitation services for all in all settings, especially schools and health-care facilities. Parallel processes should be avoided. It is also important that water, sanitation and health (WASH) matters to be dealt with within the framework of the Protocol^5^ become fully integrated in existing national processes (e.g. EU integration). A strong coordination platform is needed to guide implementation of the Protocol^5^ and action towards achievement of the WASH-related SDGs.^3^ The establishment of a national working group on the Protocol will help ensure coherence in this respect.

Recently, the Law on Protocol has been adopted by the Parliament. Montenegro’s experience in working towards achievement of the United Nations 2030 Agenda’s SDGs^3^ and ratification of the Protocol on Water and Health^5^ has the following key messages to share:

- WASH matters should be integrated within existing national policies and considered within the EU integration process. In Montenegro, the planning and accountability approach of the Protocol on Water and Health^5^ offers a practical framework for translation of the SDGs^3^ and the Ostrava Declaration on Environment and Health^7^ into tangible national targets and actions, taking the national circumstances into account.
- An inclusive multisectoral approach is essential to advancing WASH agendas at the national level. In Montenegro, the Protocol framework^5^ provides a national platform for bringing sectors together, as well as national and international stakeholders (such as the United Nations), all of which share responsibility in the water and sanitation domain.
- Political commitment is an important ingredient for progress. The engagement of parliamentary representatives will be critical for a broad acceptance and the success of the Protocol^5^ in Montenegro.
- International technical support and networks have spearheaded the Protocol ratification process. The international support provided by the Iceland Statement of the Small Countries Initiative, Ensuring safe and climate-resilient water and sanitation (2018), provides an additional boost to the process towards ratification of the Protocol.^5^

## Disclaimer

The authors alone are responsible for the views expressed in this article, and they do not necessarily represent the views, decisions or policies of the institutions with which they are affiliated.

*Conflicts of interest*: None declared.
